# Biomedical PCL Janus patch with mushroom-structured wet adhesive and PEG-coated anti-adhesive surface

**DOI:** 10.1039/d6ra06730a

**Published:** 2026-08-11

**Authors:** Shinyull Lee, Woochan Kim, Harshita Sharma, Sefali Bhakuni, Dream Kim, Jangho Kim

**Affiliations:** a Department of Convergence Biosystems Engineering, Chonnam National University Gwangju 61186 Republic of Korea rain2000@jnu.ac.kr; b Department of Rural and Biosystems Engineering, Chonnam National University Gwangju 61186 Republic of Korea; c Interdisciplinary Program in IT-Bio Convergence System, Chonnam National University Gwangju 61186 Republic of Korea; d Institute of Nano-Stem Cells Therapeutics, NANOBIOSYSTEM Co., Ltd Gwangju 61011 Republic of Korea

## Abstract

Simultaneously achieving tissue adhesion and anti-adhesion remains a fundamental challenge for biomedical patch development. Here, we developed a PCL-based Janus patch that combines wet adhesion mediated by mushroom-shaped microstructures with an anti-adhesive surface formed by UV-crosslinked PEG. The mushroom-structured surface enhanced wet adhesion, whereas the PEG-coated surface reduced nonspecific fibroblast attachment, enabling spatially separated adhesive and anti-adhesive functions within a single PCL platform.

## Abstract 2

A bioinspired PCL/PEG Janus patch enables selective tissue interfacing through mushroom structured wet adhesion and hydrophilic anti-adhesive protection.

## Graphical abstract

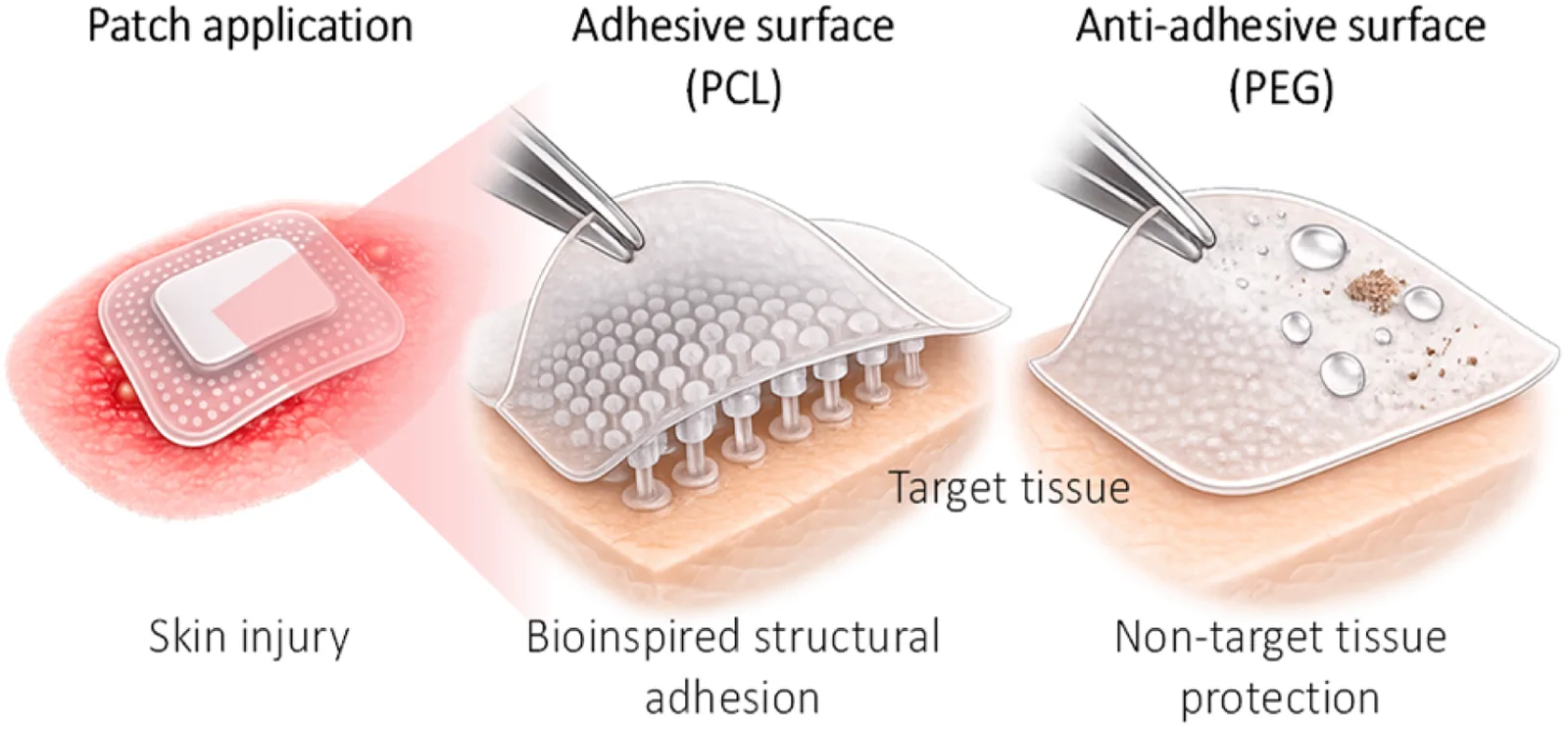

Patch-based systems represent one of the most straightforward and widely adopted formats for establishing functional interfaces between materials and biological tissues.^1–3^ Owing to their structural simplicity, patch systems have been extensively applied across diverse fields, including cosmetic delivery platforms, wound dressings, tissue-contact therapeutic systems, and implantable biomedical devices.^1–4^ Despite this conceptual simplicity, the practical design of patch interfaces often encounters a fundamental contradiction: the need to simultaneously achieve strong adhesion to the target tissue while preventing unintended adhesion to surrounding biological structures.^3,4^

These competing requirements are particularly relevant in biomedical applications such as wound management, postoperative adhesion prevention, and tissue-contact therapeutic systems, where stable fixation must coexist with isolation from surrounding tissues.^5–7^ In wound care, for instance, patches must remain firmly attached to the damaged tissue surface to maintain conformal sealing and therapeutic stability.^5^ At the same time, unwanted adhesion to neighboring tissues, surgical cavities, or biological fluids must be minimized to prevent complications such as secondary adhesion, irritation, or tissue damage during removal.^6,7^

Conventional patch systems typically address only one of these interfacial requirements, despite the clinical need for materials that simultaneously adhere to target tissues while preventing undesired attachment to surrounding organs.^5,6^ Adhesive patches provide strong fixation but frequently lead to uncontrolled secondary adhesion, residue formation, and tissue irritation upon removal.^6^ Conversely, anti-adhesive barriers effectively suppress biological attachment but lack sufficient positional stability, limiting their use in applications that require robust fixation.^7^ As a result, adhesion and anti-adhesion remain fundamentally incompatible functions in most existing biomaterial systems, and current approaches largely rely on separate materials rather than integrating both functions within a single platform.^5–7^

Janus-type materials, which spatially separate incompatible functions on opposite surfaces, provide an attractive conceptual strategy to overcome this limitation.^8–10^ However, many previously reported Janus systems rely on newly synthesized polymers, bioinspired adhesive chemistries, or multi-step fabrication strategies.^11,12^ Although these approaches demonstrate promising functionality, their reliance on complex material synthesis and fabrication processes can limit the scalability of manufacturing and regulatory translation. Consequently, most Janus systems remain material-intensive and fabrication-dependent, rather than leveraging clinically established biomaterials through simple design strategies.^9,13^ Unlike our previously reported PLGA/chitosan-based Janus patch combining mushroom-shaped adhesive and microsharp anti-adhesive topographies, the present study employs a distinct PCL-based strategy that couples mushroom-structured wet adhesion with UV-crosslinked PEG-mediated reduction in fibroblast attachment.

In this study, we demonstrate that the combination of mushroom-shaped microstructural engineering and polyethylene glycol (PEG) surface modification can transform polycaprolactone (PCL) into a selective tissue interface. By introducing a Janus architecture that combines microstructure-driven adhesion with a PEG-coated anti-adhesive surface, this study transforms an otherwise passive polymer into a programmable tissue-selective platform. Although PCL is widely used in biomedical devices because of its biocompatibility and clinical familiarity, its relatively inert surface properties make it difficult to achieve controlled and selective tissue interactions.^14,15^ We hypothesized that the limited wet adhesion observed in conventional PCL patches arises primarily from the absence of an appropriate interfacial architecture rather than intrinsic material limitations. Guided by this hypothesis, we designed a dual-surface patch in which one side incorporates mushroom-inspired microstructures to generate strong wet adhesion, while the opposite surface is modified with PEG to create a hydrophilic anti-adhesive barrier.^16,17^

The overall design concept of the selective tissue interface (STI) Janus patch is illustrated in Fig. 1A. In this configuration, the microstructured PCL surface faces the wound bed to promote stable tissue adhesion and conformal sealing, whereas the PEG-modified surface faces the surrounding biological environment to suppress nonspecific interactions and prevent secondary adhesion.^18–20^ An enlarged schematic of the tissue interface further highlights this spatially resolved functionality, where microstructure-driven mechanical interlocking occurs on the PCL side, while the PEG layer provides anti-adhesive protection on the outward-facing surface (Fig. 1B).

**Fig. 1:**
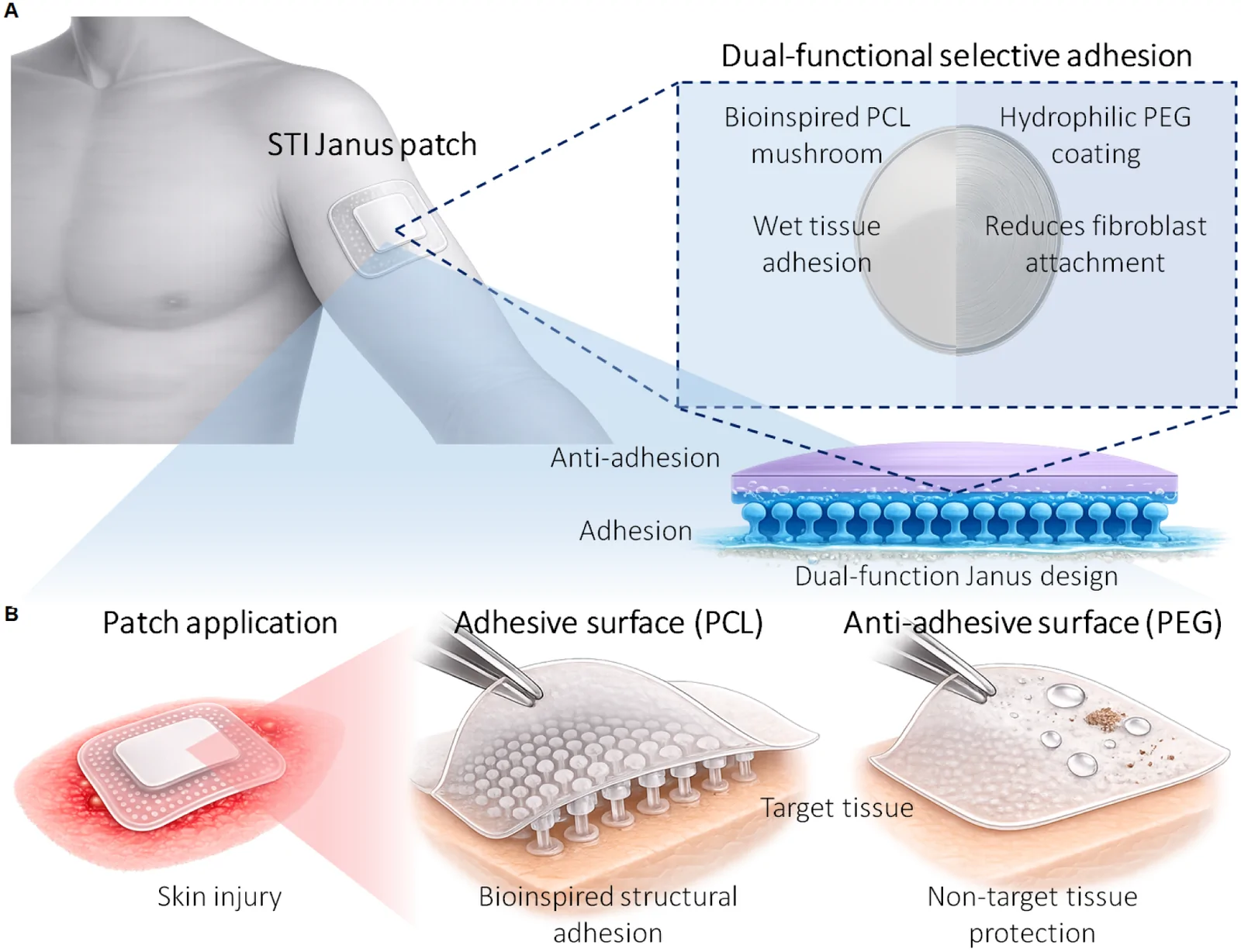
Design concept of the selective tissue interface (STI) Janus patch. (A) Schematic illustration of the dual-surface PCL-PEG Janus patch, comprising a mushroom-structured PCL surface for wet adhesion to target tissue and an opposing PEG-coated surface designed to reduce unintended cellular attachment. (B) Enlarged illustration showing the spatial separation of microstructure-mediated wet adhesion on the PCL side and PEG-mediated reduction in cellular attachment on the opposite side.

To realize this design, the adhesive surface of the patch was fabricated by replicating mushroom-shaped microstructures on the PCL surface by micromolding. The mushroom-shaped geometry was selected based on previous bioinspired adhesion studies demonstrating superior adhesion under wet conditions.^21–25^ The mushroom-shaped architecture increases the effective contact area and promotes mechanical engagement with the tissue surface, thereby enhancing adhesion without requiring additional adhesive chemistries.^22,23^ Importantly, the overhanging cap geometry distributes interfacial stress and resists detachment, and has been widely recognized as one of the most effective microstructural designs for achieving strong adhesion in soft and wet environments.^21,22,25,26^

Importantly, the microstructures were formed uniformly across the PCL film without compromising its structural continuity or mechanical integrity. The resulting microstructured surface maintained the inherent flexibility of the polymer film while introducing an architecture capable of generating strong interfacial adhesion. As summarized in Fig. 2, the dual-surface PCL-PEG Janus construct was successfully fabricated and structurally characterized. A cross-sectional SEM image of the PCL-PEG Janus patch and a magnified view of the fabricated mushroom-shaped microstructures are provided in Fig. S3, confirming the successful replication of the cap-and-stem morphology.

**Fig. 2:**
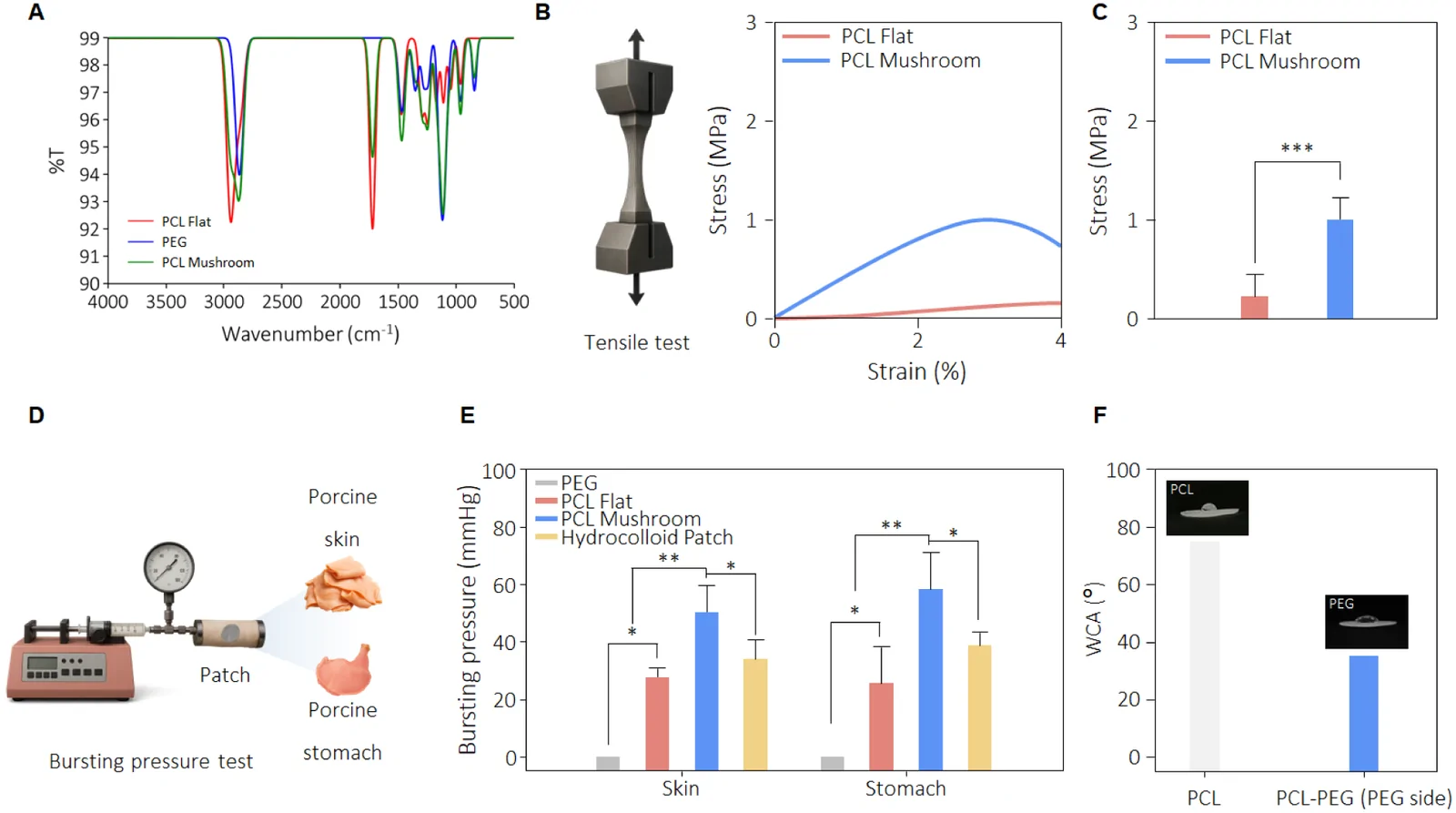
Structural characterization and functional performance of the PCL-PEG Janus patch. (A) FT-IR spectra of PCL Flat, PEG, and PCL Mushroom samples showing their characteristic absorption bands. (B) Tensile-test configuration and representative stress–strain curves of PCL Flat and PCL Mushroom samples. (C) Comparison of tensile strength between PCL flat and PCL mushroom samples. (D) Schematic illustration of the bursting-pressure test using *ex vivo* porcine skin and stomach tissues. (E) Bursting pressures measured for the PEG-coated side, PCL flat, PCL mushroom, and a commercial hydrocolloid patch on porcine skin and stomach tissues. (F) Water contact angles of PCL and PCL-PEG (PEG side), showing increased surface hydrophilicity associated with PEG modification.

Fourier-transform infrared (FT-IR) spectroscopy confirmed the coexistence of characteristic PCL and PEG spectral features, indicating the successful formation of the Janus configuration (Fig. 2A). Mechanical testing showed that the microstructured PCL Mushroom patch retained sufficient tensile strength while exhibiting enhanced elongation compared with the PCL Flat control (Fig. 2B and C). These results indicate that microstructuring preserves mechanical robustness while improving flexibility, which may facilitate conformal contact with irregular tissue surfaces.

Beyond structural integrity, the Janus design directly translates into improved functional performance. In burst-pressure tests using circular defects created in porcine skin and stomach tissues, the patch provided effective sealing performance and maintained conformal contact under physiologically relevant stress conditions (Fig. 2D and E). These results indicate that the microstructured adhesive surface can sustain stable tissue contact while resisting fluid leakage across the damaged tissue interface.

An anti-adhesive surface was generated by UV-crosslinking PEG onto the opposite side of the PCL film, forming a chemically stabilized hydrophilic layer. Water contact angle measurements verified the increased hydrophilicity associated with PEG surface modification and were not interpreted as direct evidence of anti-adhesion. Direct evaluation using a 24 h NIH3T3 fibroblast attachment assay demonstrated a significantly lower density of attached cells on the PEG-coated surface than on the PCL Flat and PCL Mushroom surfaces under the tested *in vitro* conditions (Fig. 5).

When integrated into a single construct, the Janus patch provides spatially resolved functionality: strong tissue adhesion on the microstructured PCL side and biological isolation on the PEG-modified side. This dual-function configuration directly addresses the central limitation of conventional patch systems, which typically cannot provide stable fixation and anti-adhesion within a single platform.

The adhesive performance of the microstructured PCL surface was further evaluated across a range of representative substrates under dry and wet conditions. Under both dry and wet conditions, the PCL Mushroom surface exhibited significantly greater adhesion than the PCL Flat control, while the commercial hydrocolloid patch showed comparatively lower adhesion. These results demonstrate that the enhanced adhesive performance originates from the mushroom-shaped microarchitecture rather than from the intrinsic properties of the PCL material (Fig. 3A–D).

**Fig. 3:**
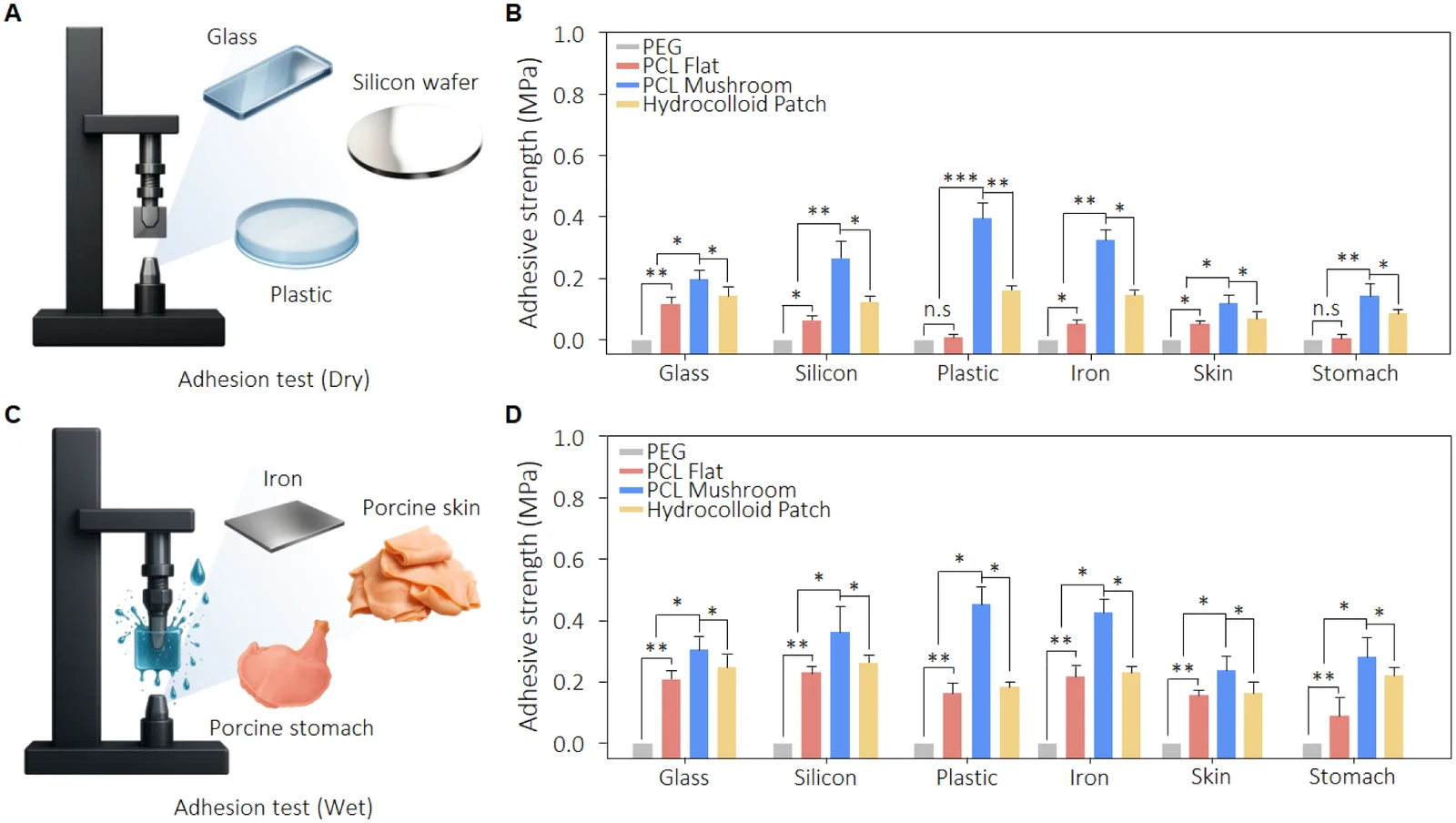
Comparison of adhesion performance under dry and wet conditions. (A) Schematic illustration of the dry adhesion test using representative substrates. (B) Adhesive strengths of PCL-PEG (PEG side), PCL flat, PCL mushroom, and a commercial hydrocolloid patch on glass, silicon wafer, plastic, iron, porcine skin, and porcine stomach under dry conditions. (C) Schematic illustration of the wet adhesion test using PBS-hydrated substrates. (D) Adhesive strengths of the four groups under wet conditions. The greater adhesion of PCL Mushroom compared with PCL Flat under both conditions supports the contribution of the mushroom-shaped microarchitecture to adhesion enhancement under the tested conditions.

Based on previous bioinspired adhesion studies, this architecture-dependent enhancement may be associated with increased effective interfacial contact and redistribution of detachment stresses by the mushroom-shaped terminal geometry. However, the individual contributions of suction, capillary forces, and viscoelastic dissipation were not separately resolved in the present study.

These findings demonstrate that the mushroom-shaped microstructures significantly enhanced the adhesion of the PCL surface under both dry and wet conditions. Compared with a commercial hydrocolloid dressing, PCL Mushroom exhibited greater adhesion on the representative substrates tested. We next evaluated the *in vitro* cytocompatibility of the Janus platform to determine whether structural asymmetry and PEG modification affected fibroblast responses. As illustrated in Fig. 4A, disc-shaped PCL Flat and PCL-PEG substrates were used as cell culture platforms.

**Fig. 4:**
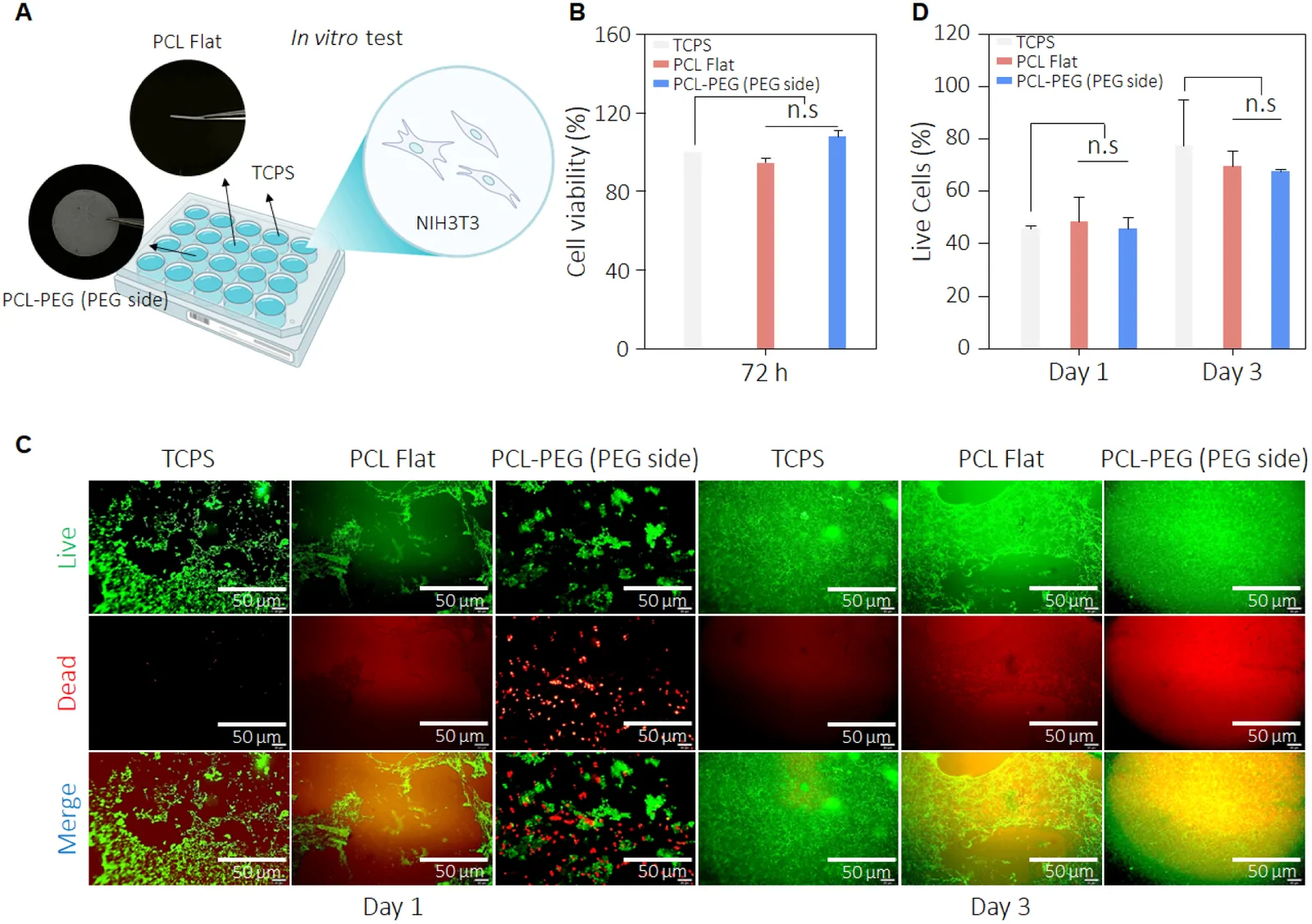
*In vitro* cytocompatibility evaluation of the PCL-PEG Janus patch. (A) Schematic illustration of NIH3T3 fibroblast culture on tissue-culture-treated polystyrene (TCPS), PCL flat, and PCL-PEG (PEG side) surfaces. (B) Metabolic cell viability after 72 h of culture. (C) Representative live/dead fluorescence images obtained after 1 and 3 days of culture. Live and dead cells are shown in green and red, respectively. Scale bars: 50 µm. (D) Quantification of the percentage of live cells on days 1 and 3.

Metabolic activity analysis demonstrated that NIH3T3 fibroblasts maintained high viability on the Janus patch surfaces throughout the culture period (Fig. 4B–D). Live/dead staining further confirmed robust cell survival, indicating that the structural modification and PEG surface treatment did not introduce cytotoxic effects.

To evaluate water vapor transport, the water vapor transmission rate (WVTR) of the patch materials was measured over 24 h at 37 °C and 50% relative humidity (Fig. S5). No statistically significant differences were observed among the PCL Flat, PCL Mushroom, and PCL-PEG Janus groups, indicating that mushroom-shaped microstructuring and PEG surface modification did not significantly alter water vapor transmission under the tested conditions. A commercial hydrocolloid dressing was included as a reference.

To directly evaluate fibroblast attachment on the PEG-coated surface, NIH3T3 fibroblasts were cultured for 24 h on tissue-culture-treated polystyrene (TCPS), PCL Flat, PCL-PEG (PEG side), and PCL mushroom surfaces (Fig. 5A). Fluorescence imaging revealed fewer attached cells on the PCL-PEG surface than on the unmodified PCL surfaces (Fig. 5B). Quantitative analysis confirmed that the density of attached cells on the PCL-PEG surface was significantly lower than the corresponding densities on the PCL flat and PCL mushroom surfaces, demonstrating reduced fibroblast attachment under the tested *in vitro* conditions (Fig. 5C). RT-qPCR was additionally performed to characterize the expression of selected ECM- and repair-associated genes in the attached cell population (Fig. 5D); however, these data were not used as direct evidence of anti-adhesive performance.

**Fig. 5:**
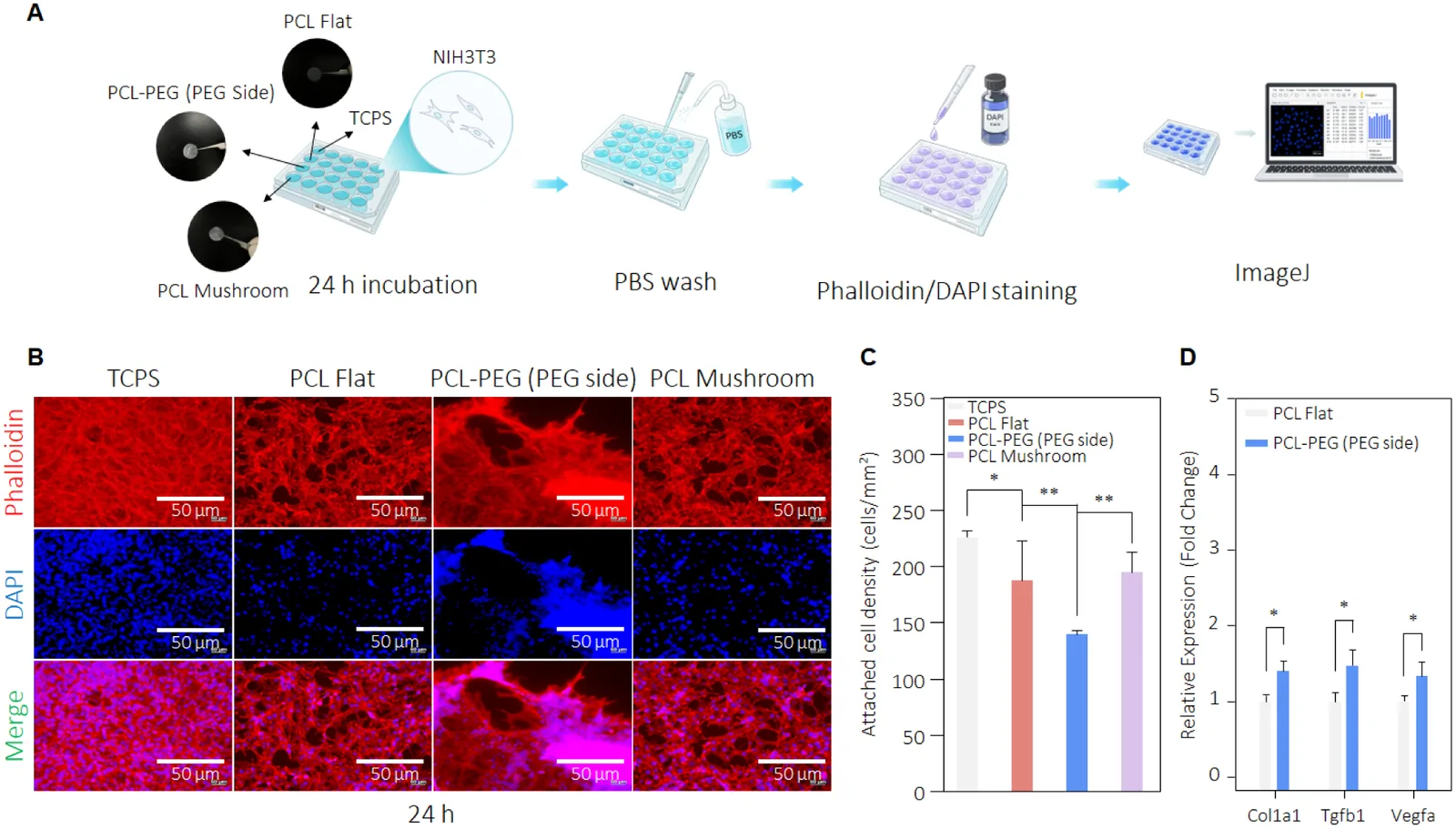
*In vitro* evaluation of NIH3T3 fibroblast attachment to the PEG-coated surface and characterization of the remaining attached cells. (A) Schematic workflow of the 24 h cell-attachment assay, including PBS washing, fluorescence staining, and image analysis. (B) Representative fluorescence images of cells remaining attached to TCPS, PCL Flat, PCL-PEG (PEG side), and PCL mushroom surfaces after PBS washing. F-actin and nuclei were stained with phalloidin (red) and DAPI (blue), respectively. Scale bars: 50 µm. (C) Attached cell density quantified by counting DAPI-positive nuclei and expressed as cells mm^−2^. (D) Relative mRNA expression of Col1a1, Tgfb1, and Vegfa in cells remaining attached to PCL Flat and PCL-PEG (PEG side). Expression was normalized to Gapdh and calculated using the 2^−^ΔΔCt method, with PCL Flat used as the calibrator. Data are presented as mean ± SD (*n* = 6 independent experiments per group). *p < 0.05 and **p < 0.01. Detailed experimental and statistical procedures are provided in the SI.

The present study demonstrates a PCL-based Janus patch that spatially integrates a mushroom-structured adhesive surface with an opposing surface coated with UV-crosslinked PEG. Relative to PCL flat, the PCL mushroom surface exhibited significantly greater adhesion under dry and wet conditions, whereas the PEG-coated surface exhibited significantly lower NIH3T3 fibroblast attachment after 24 h than the PCL Flat and PCL Mushroom surfaces. The principal contribution of this work is the integration of mushroom-structure-mediated adhesion and PEG-mediated reduction in fibroblast attachment within a single PCL-based construct.

## Author contributions

J. K. conceived and supervised the study. S. L. performed the experiments, analyzed the data, and wrote the original draft of the manuscript. W. K., H. S., S. B., and D. K. contributed to the investigation and data interpretation. All authors reviewed and edited the manuscript and approved the final version of the manuscript.

## Conflicts of interest

There are no conflicts to declare.

## Supplementary Material

RA-016-D6RA06730A-s001

## Data Availability

The data supporting this article are included in the supplementary information (SI). Supplementary information: detailed experimental methods and supplementary figures describing the fabrication process, patch morphology, NIH3T3 fibroblast fluorescence analysis, and water vapor transmission rate (WVTR) of the PCL–PEG Janus patch. See DOI: https://doi.org/10.1039/d6ra06730a.

## References

[cit1] Pastore M. N., Kalia Y. N., Horstmann M., Roberts M. S. (2015). Br. J. Pharmacol..

[cit2] Wang Y., Wang Y., Xu X., Zhang Y., Li Y., Zhang H., Gui Y., Cai W. (2021). ACS Nano.

[cit3] Wong S. H. D., Deen G. R., Bates J. S., Maiti C., Lam C. Y. K., Pachauri A., AlAnsari R., Bělský P., Yoon J., Dodda J. M. (2023). Adv. Funct. Mater..

[cit4] Deng X., Gould M., Ali M. A. (2022). J. Biomed. Mater. Res., Part B.

[cit5] Nie Z., Petukhova A., Kumacheva E. (2010). Nat. Nanotechnol..

[cit6] Zhang J., Seeger S. (2011). Adv. Mater..

[cit7] Jiang Y., Chen L., Zhu C., Zhang L., Shi F. (2016). Adv. Mater..

[cit8] Gorb S., Varenberg M., Peressadko A., Tuma J. (2007). J. R. Soc. Interface.

[cit9] Aksak B., Murphy M., Sitti M. (2007). Bioinspiration Biomimetics.

[cit10] Kim S., Sitti M. (2006). Appl. Phys. Lett..

[cit11] Lee J. M., Lee S. H., Kim H. J., Park J., Lee J. H. (2014). Biomaterials.

[cit12] Sun Y., Jiang L., Liu Y., Wu J. (2017). ACS Appl. Mater. Interfaces.

[cit13] Dalsin J. L., Hu B. H., Lee B. P., Messersmith P. B. (2003). J. Am. Chem. Soc..

[cit14] Cao Z., Jiang S. (2007). Langmuir.

[cit15] Chen Y., Yang H., Zhang J., Zhang Y., Jiang L. (2018). Adv. Funct. Mater..

[cit16] Zhao Y., Xie Z., Gu H., Jin C., Jiang L. (2012). Adv. Mater..

[cit17] Yuk H., Zhang T., Parada G. A., Liu X., Zhao X. (2016). Nat. Mater..

[cit18] Lee H., Lee B. P., Messersmith P. B. (2007). Nature.

[cit19] Liu Y., He K., Chen G., Leow W. R., Chen X. (2017). Chem. Rev..

[cit20] Arzt E., Gorb S., Spolenak R. (2003). Proc. Natl. Acad. Sci. U. S. A..

[cit21] Tian H., Zhao Y., Li Z., Wang Y., Chen X. (2024). Adv. Sci..

[cit22] Tian H., Wang D., Zhang Y., Jiang Y., Liu T., Li X., Wang C., Chen X., Shao J. (2022). Nat. Commun..

[cit23] Li R., Chen Y., Xu Z., Jiang L. (2024). Int. J. Solids Struct..

[cit24] Liu F., Zhang J., Shao Y., Chen X. (2025). Microsyst. Nanoeng..

[cit25] Lee S., Kim W., Sharma H., Safdar M., Kim D., Park C., Kim J. (2025). Nano Lett..

[cit26] Lee S., Park S., Sharma H., Safdar M., Park S., Kim W., Kim D., Oh J., Kim J. (2024). ACS Agric. Sci. Technol..

