# Supplementary material for: Biomedical PCL Janus patch with mushroom-structured wet adhesive and PEG-coated anti-adhesive surface
Source: RSC Adv. 2026 Aug 11;16(41):48090–4. doi: 10.1039/d6ra06730a (PMC13458451; doi:10.1039/d6ra06730a)
Supplement: Supplementary file 1 [file RA-016-D6RA06730A-s001.pdf]

Supplementary Information (SI) for RSC Advances.
This journal is © The Royal Society of Chemistry 2026

Supplementary Information

Biomedical PCL Janus Patch with Mushroom-Structured Wet Adhesive and PEG-coated Anti-Adhesive Surface

Shinyull Lee,[abc] *Woochan Kim,*[abc] *Harshita Sharma,*[abc] *Sefali Bhakuni,*[abc] *Dream Kim,*[abc] *and Jangho Kim* [*abcd]

[a] Department of Convergence Biosystems Engineering, Chonnam National University, Gwangju, 61186, Republic of Korea.

[b] Department of Rural and Biosystems Engineering, Chonnam National University, Gwangju 61186, Republic of Korea.

[c] Interdisciplinary Program in IT-Bio Convergence System, Chonnam National University, Gwangju 61186, Republic of Korea.

[d] Institute of Nano-Stem Cells Therapeutics, NANOBIOSYSTEM Co., Ltd, Gwangju 61011, Republic of Korea.

[*] Corresponding author: J. Kim (rain2000@jnu.ac.kr)

Experimental section

Materials

Polycaprolactone (PCL, Mw ~80,000), polyethylene glycol diacrylate (PEGDA), photoinitiator, phosphate-buffered saline (PBS), Dulbecco's Modified Eagle Medium (DMEM), fetal bovine serum (FBS), penicillin–streptomycin, calcein-AM, ethidium homodimer-1, phalloidin, DAPI, and other analytical-grade reagents were purchased from commercial suppliers and used as received without further purification.

NIH3T3 fibroblasts were cultured in DMEM supplemented with 10% FBS and 1% penicillin–streptomycin at 37 °C in a humidified atmosphere containing 5% CO_2.

Fabrication of the Mushroom-Structured PCL Surface

The adhesive PCL surface containing mushroom-shaped microstructures was fabricated using a soft lithography-based micro-molding process. Silicon-on-insulator (SOI) wafers were treated with hydrophobic trichloro(1H,1H,2H,2H-perfluorooctyl) silane to facilitate mold release.

A poly(dimethylsiloxane) (PDMS) precursor mixture consisting of base and curing agent (10:1, w/w) was thoroughly mixed and degassed under vacuum for 100 min to remove trapped air bubbles. The degassed PDMS precursor was cast onto polyurethane acrylate (PUA) replica molds and further vacuum-treated to ensure complete filling of the microcavities.

After curing at 50 °C for 12 h, the PDMS molds were carefully detached after cooling. PCL solution was subsequently cast onto the prepared molds and dried to obtain mushroom-structured PCL films. The resulting adhesive microstructures exhibited diameters of approximately 6.43 μm

and heights of approximately 16.8 μm. Flat PCL films without microstructures were fabricated under identical conditions and used as controls.

Fabrication of the PCL/PEG Janus Patch

To generate the anti-adhesive interface, a PEGDA precursor solution containing photoinitiator was coated onto the opposite side of the mushroom-structured PCL film.

UV irradiation was subsequently applied to induce PEG crosslinking and form a chemically stabilized hydrophilic PEG layer. The resulting Janus construct consisted of a mushroom-structured adhesive PCL surface and a PEG-coated anti-adhesive surface integrated into a single platform.

Fourier-Transform Infrared (FT-IR) Spectroscopy

FT-IR spectroscopy was performed using a PerkinElmer Spectrum IR instrument equipped with an ATR MIRacle Diamond Frontier accessory under ambient conditions (~25 °C). Each sample was directly placed onto the ATR crystal, and spectra were collected over a wavenumber range of 4000–450 cm^{-1} at a resolution of 4 cm^{-1} with 16 scans per sample.

Characteristic absorption peaks corresponding to PCL and PEG functional groups were analyzed to confirm the successful formation of the Janus structure.

Scanning Electron Microscopy (SEM)

For SEM analysis, samples were mounted onto aluminum stubs using conductive carbon tape and sputter-coated with gold (~10 nm thickness). SEM images were obtained using a field-emission scanning electron microscope operated at an acceleration voltage of 10 kV.

The morphology, structural fidelity, and uniformity of the mushroom-shaped microstructures were analyzed using high-resolution SEM imaging.

Mechanical Characterization

Mechanical properties were evaluated using a universal testing machine at a strain rate of 10 mm min^{-1}. Stress–strain curves were obtained for both PCL Flat and PCL Mushroom samples.

Tensile strength and elongation at break were analyzed to evaluate the influence of microstructural engineering on the mechanical performance and flexibility of the PCL films.

Water Contact Angle Measurement

Static water contact angles were measured using a contact angle analyzer to evaluate surface wettability. A droplet of deionized water was gently deposited onto each sample surface, and the contact angle was immediately recorded.

Measurements were performed on both the PCL surface and PEG-coated surface to confirm the increased hydrophilicity of the PEG-modified interface.

Adhesion Test Under Dry and Wet Conditions

The adhesion performance of PCL Flat, PCL Mushroom, and a commercial hydrocolloid patch was evaluated on glass, silicon wafer, plastic, iron, ex vivo porcine skin, and porcine stomach tissue under dry and wet conditions. PCL Flat and PCL Mushroom samples were prepared from the same PCL material and tested using identical contact areas. For hydrated testing, each substrate was uniformly wetted with PBS before patch application. The adhesive surface was applied to the substrate under the same preload for 30 s and subsequently detached using a force-testing system.

The maximum detachment force was normalized to the contact area to calculate adhesion strength. All groups were evaluated using identical testing conditions.

Bursting Pressure Test

Bursting pressure tests were performed using porcine skin and stomach tissues containing circular defects. The Janus patch was applied over the defect region, and internal pressure was gradually increased until fluid leakage occurred.

The maximum pressure sustained before leakage was recorded as the bursting pressure.

Preparation of Porcine Skin and Stomach Tissues

Porcine skin and stomach tissues were obtained from a local supplier and transported to the laboratory on ice. Hair on the skin tissues was carefully removed using disposable razors, followed by washing with PBS.

Residual fat was removed by alternating immersion in ethanol and PBS for one week with daily solution replacement. The tissues were subsequently cut into uniform 5 cm × 5 cm sections and equilibrated to room temperature prior to adhesion and mechanical testing.

In Vitro Cytocompatibility Assay

Janus patches were sterilized under UV irradiation for 30 min prior to cell culture experiments. NIH3T3 fibroblasts were seeded onto the sample surfaces at a density of 5×10^4 cells cm^{-2} and cultured under standard conditions.

Cell viability and cytocompatibility were evaluated using Live/Dead staining and metabolic activity assays after predetermined culture periods.

Live/Dead Staining

After culture, cells were stained using calcein-AM and ethidium homodimer-1 to visualize live and dead cells, respectively. Fluorescence images were acquired using confocal laser scanning microscopy.

Cell viability was quantitatively analyzed based on fluorescence intensity and live/dead cell ratios.

Fluorescence Imaging of Cytoskeletal Organization

For cytoskeletal staining, cells were fixed with paraformaldehyde and stained with phalloidin for F-actin visualization and DAPI for nuclear staining.

Fluorescence images were obtained using confocal microscopy to evaluate cell spreading, cytoskeletal organization, and cell–material interactions on the patch surfaces.

RT-qPCR Analysis

NIH3T3 fibroblasts were cultured on PCL Flat and PCL-PEG (PEG side) surfaces for 24 h. After gentle washing twice with phosphate-buffered saline (PBS), total RNA was extracted from the remaining attached cells, and complementary DNA (cDNA) was synthesized by reverse transcription. RT-qPCR was performed to evaluate the expression of the ECM- and repair-associated genes *Col1a1*, *Tgfb1*, and *Vegfa*. Gene expression was normalized to *Gapdh* and calculated using the $2^{-\Delta\Delta Ct}$ method, with the mean expression level of the PCL Flat group set to

1 for each gene. Statistical comparisons were performed using ΔCt values with a two-sided unpaired Student's t-test, followed by Holm–Šídák correction for the three gene comparisons. Data are presented as mean ± SD from six independent experiments per group ($n = 6$). These data were used to characterize the remaining attached cell population and not as direct evidence of anti-adhesive performance.

Statistical Analysis

All quantitative data are presented as mean ± standard deviation. Statistical analyses were performed using GraphPad Prism software.

Statistical significance was determined using one-way ANOVA, and differences were considered statistically significant at $p < 0.05$.

NIH3T3 Fibroblast Attachment Assay

To directly evaluate fibroblast attachment to the PEG-coated surface, NIH3T3 cell attachment was compared among tissue-culture-treated polystyrene (TCPS), PCL Flat, PCL-PEG (PEG side), and PCL Mushroom surfaces. The samples were prepared and sterilized as described above. NIH3T3 fibroblasts were seeded onto each surface at a density of 5×10^4 cells cm^{-2} and cultured for 24 h.

After incubation, the samples were gently washed twice with phosphate-buffered saline (PBS) to remove non-adherent cells. The remaining attached cells were fixed, permeabilized, and stained with phalloidin and DAPI as described above. Fluorescence images were acquired under identical imaging conditions, and at least five randomly selected, non-overlapping fields were analyzed for each sample.

Attached cells were quantified by counting DAPI-positive nuclei using ImageJ software (National Institutes of Health, Bethesda, MD, USA). Identical thresholding and particle-analysis settings were applied to all groups. Attached cell density was calculated as the number of DAPI-positive nuclei divided by the imaged area (mm^2). The values obtained from at least five fields were averaged to generate one independent value for each sample, and individual fields were not treated as independent replicates. Six independent experiments were performed per group (n = 6). Data were analyzed using one-way ANOVA followed by Dunnett's multiple-comparisons test, with the PCL-PEG group used as the reference group. A value of $p < 0.05$ was considered statistically significant.

Water Vapor Transmission Rate (WVTR) Measurement

WVTR was measured using a gravimetric method. PCL Flat, PCL Mushroom, PCL-PEG Janus, and commercial hydrocolloid samples were sealed over glass vials containing distilled water. The exposed area was defined by a 20 mm-diameter opening. The assemblies were maintained at 37°C and 50% relative humidity, and their masses were recorded at 0, 12, and 24 h. WVTR was calculated from the mass-loss rate per exposed area and expressed as $g\ m^{-2}\ day^{-1}$. Three independent samples were analyzed per group (n = 3), and data are presented as mean ± SD. Statistical differences were evaluated using one-way ANOVA followed by Tukey's multiple-comparisons test.

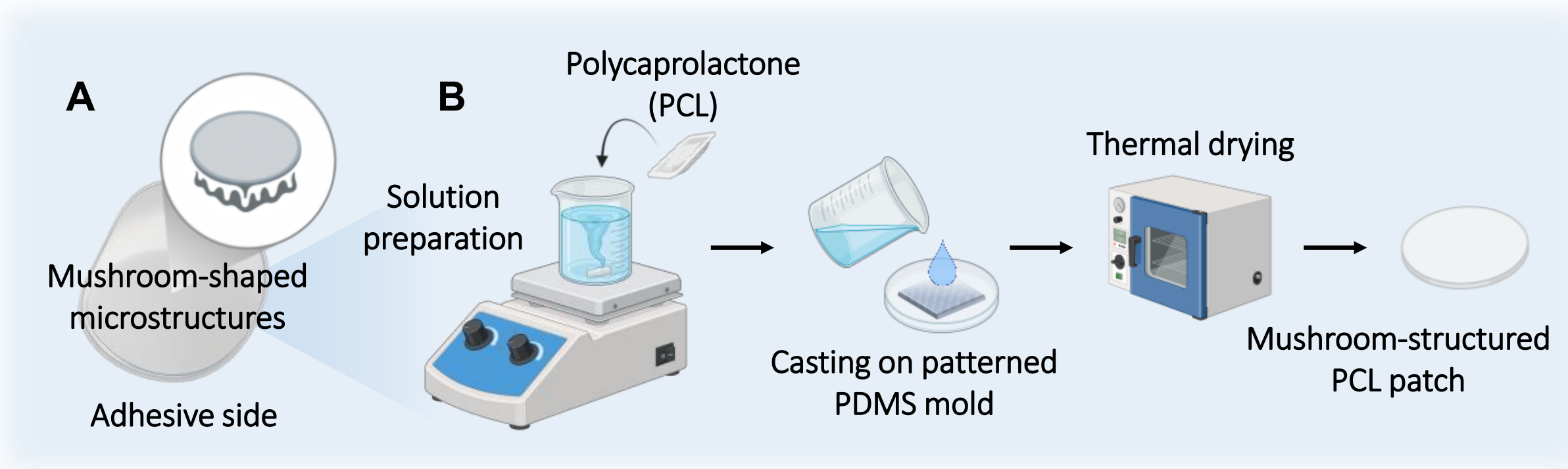

Figure S1. Mushroom-structured adhesive surface and fabrication of the PCL layer. (A) Schematic illustration of the mushroom-structured PCL adhesive surface. (B) Fabrication process of the PCL layer. A PCL solution was cast onto a patterned PDMS mold and thermally dried to obtain a PCL patch bearing mushroom-shaped microstructures.

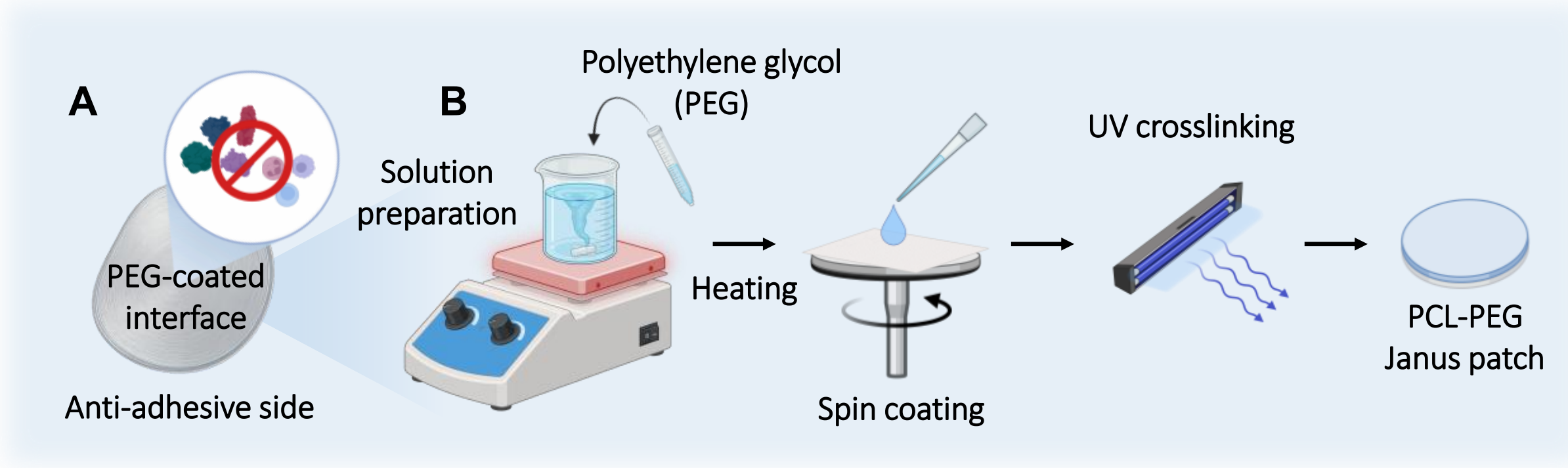

Figure S2. PEG-coated surface and fabrication of the PCL-PEG Janus patch. (A) Schematic illustration of the PEG-coated surface designed to reduce fibroblast attachment. (B) Fabrication process of the PEG-coated surface. A PEGDA solution was applied to the flat surface opposite the mushroom-structured PCL surface, spin-coated, and UV-crosslinked to form the PCL-PEG Janus patch.

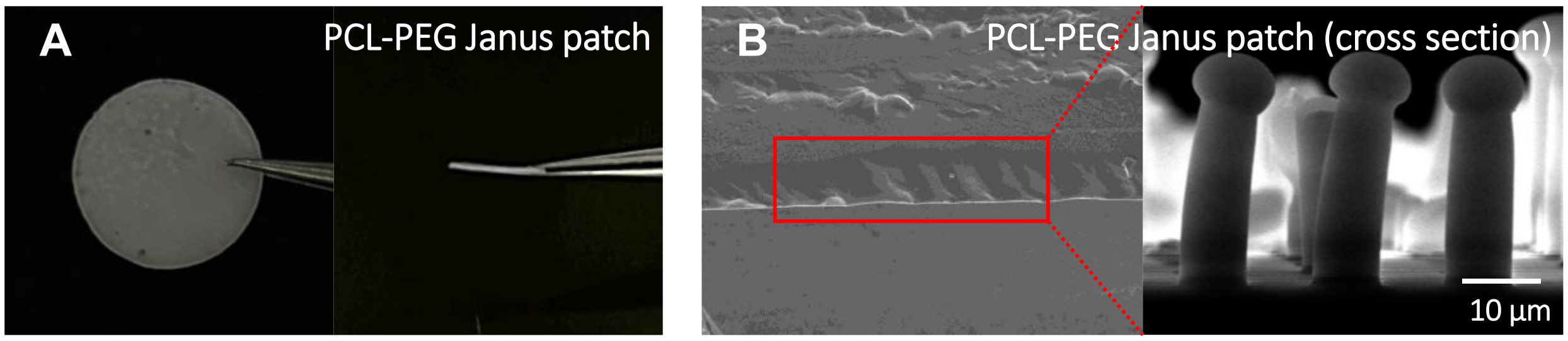

Figure S3. Macroscopic appearance and cross-sectional morphology of the PCL–PEG Janus patch. (A) Representative photographs of the circular PCL–PEG Janus patch and its bent side profile during handling with tweezers, demonstrating its macroscopic flexibility. (B) Cross-sectional SEM image of the PCL–PEG Janus patch and a magnified view of the red-boxed region, clearly showing the mushroom-shaped microstructures on the PCL adhesive surface. The red dashed lines indicate the magnified region. Scale bar in the magnified SEM image: 10 µm.

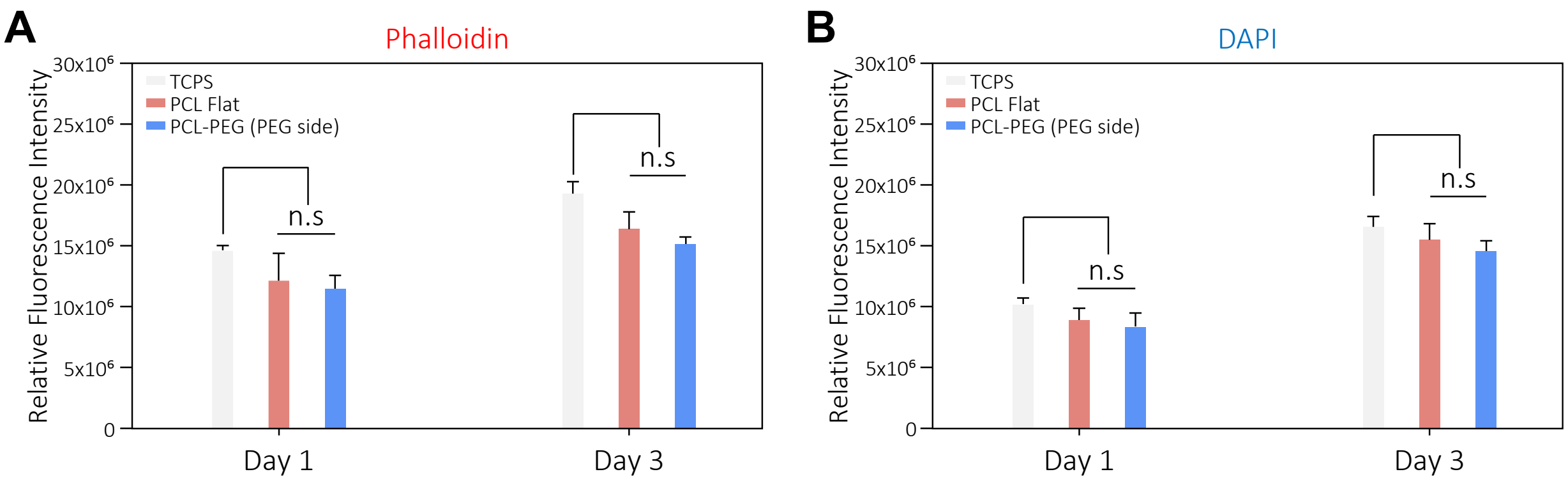

Figure S4. Quantitative analysis of cytoskeletal organization and nuclear fluorescence intensity of NIH3T3 fibroblasts. (A) Relative phalloidin fluorescence intensity of NIH3T3 fibroblasts cultured on TCPS, PCL Flat, and PCL-PEG (PEG side) surfaces after 1 and 3 days of culture. (B) Relative DAPI fluorescence intensity of NIH3T3 fibroblasts cultured on TCPS, PCL Flat, and PCL-PEG (PEG side) surfaces after 1 and 3 days of culture. Data are presented as mean ± SD (n = 3).

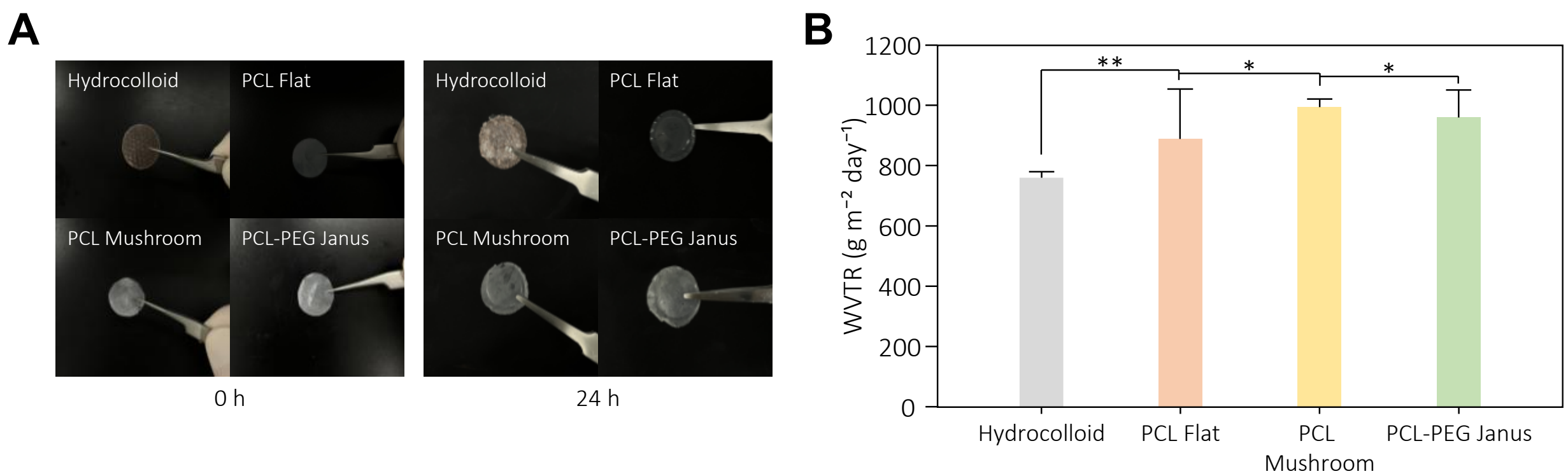

Figure S5. Water vapor transmission rate (WVTR) of different patch materials. (A) Representative photographs of the WVTR test setup at 0 and 24 h. (B) WVTR values (g m^{-2} day^{-1}) of PCL Flat, PCL Mushroom, PCL-PEG Janus, and a commercial hydrocolloid dressing measured for 24 h under controlled conditions of 37 °C and 50% relative humidity. Data are presented as mean ± SD from three independent samples per group (n = 3). Statistical differences among groups were analyzed using one-way ANOVA followed by Tukey's multiple-comparisons test ($p < 0.05$, *$p < 0.01$, and **$p < 0.001$; ns, not significant).
